# Supplementary figures and images for: Sick of news? Television news exposure, collective stressful events and headache related emergency department visits
Source: PLoS One. 2021 Apr 8;16(4):e0249749. doi: 10.1371/journal.pone.0249749 (PMC8031395; doi:10.1371/journal.pone.0249749)

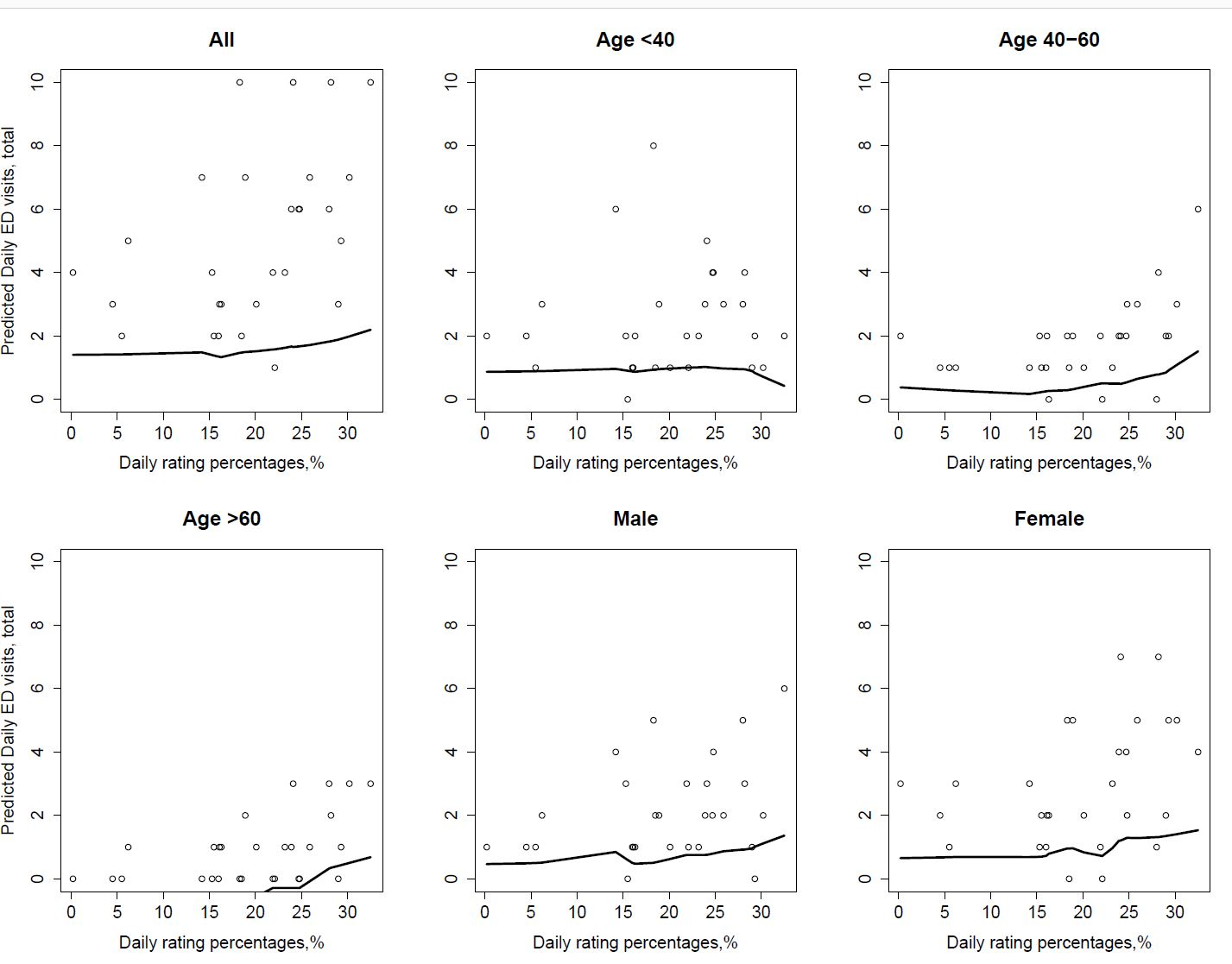

Supplement: S1 Fig — Relative Risk (RR) and 95% Confidence Interval for ED visits per increase in 5 units of daily rating percentages. Results of the separated Poisson regression models, for study period 2002–2012, adjusted for public holidays. (TIF) [file pone.0249749.s001.tif]

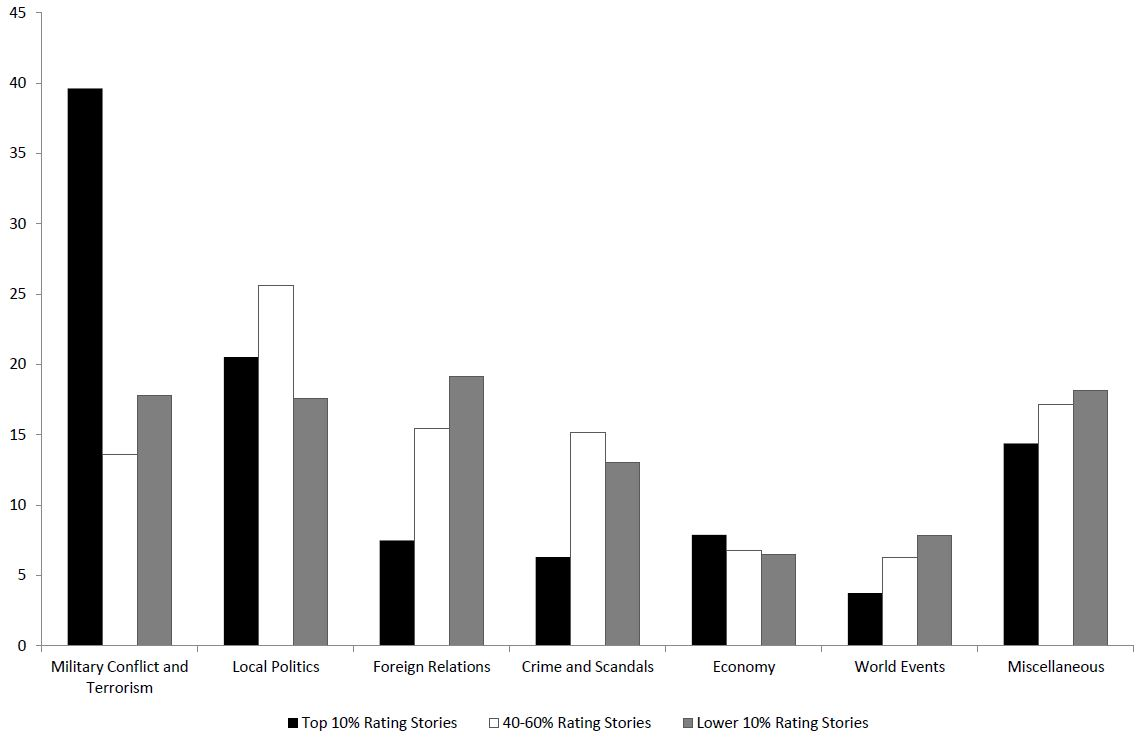

Supplement: S2 Fig — (TIF) [file pone.0249749.s002.tif]
