# Supplementary material for: Sick of news? Television news exposure, collective stressful events and headache related emergency department visits
Source: PLoS One. 2021 Apr 8;16(4):e0249749. doi: 10.1371/journal.pone.0249749 (PMC8031395; doi:10.1371/journal.pone.0249749)
Supplement: S1 Table — (DOCX) [file pone.0249749.s003.docx]

**S1 Table**

|  | **RR*** | **95%CI** |
| --- | --- | --- |
| Foreign Relations | 0.949 | 0.839-1.074 |
| Politics | 1.006 | 0.919-1.101 |
| Miscellaneous | 1.008 | 0.935-1.087 |
| World Events | 0.960 | 0.866-1.063 |
| Crime Scandals | 0.956 | 0.869-1.051 |
| Economy | 0.915 | 0.788-1.064 |
| Military Conflict and Terrorism | 0.962 | 0.885-1.047 |

**Relative Risk (RR) and 95% Confidence Interval for increase in ED visits the following day per increase in 5 units of daily rating percentages. Results of the separated Poisson regression models, for study period 2008-2012, adjusted for public holidays.*
