# Supplementary material for: Sick of news? Television news exposure, collective stressful events and headache related emergency department visits
Source: PLoS One. 2021 Apr 8;16(4):e0249749. doi: 10.1371/journal.pone.0249749 (PMC8031395; doi:10.1371/journal.pone.0249749)
Supplement: Attachment — Submitted filename: Response to Reviewers R2 Feb 2021.docx [file pone.0249749.s004.docx]

February 20th, 2021

Manuscript #: PONE-D-20-00517

Entitled; Sick of news? Television news exposure and headache related emergency department visits

Dear Dr. Joerg Heber, PhD
Editor in chief, PLOS ONE

Please see our revised submission of the manuscript entitled "Sick of news? Television news exposure and headache related emergency department visits ". We have carefully reviewed the comments of the reviewers, and would like to thank them for their thoughtful suggestions, which we think have substantially improved the quality of our manuscript. We have addressed the comments below in this letter, and in the revised manuscript attached. Please note, we have submitted both the revised version with the ‘track changes’ mode, as well as a ‘clean’ copy.

We sincerely hope the revisions made will meet your approval.

Looking forward to hearing from you,

Israel Waismel-Manor, PhD

Corresponding author

Reviewer #3: The authors followed the reviewer's suggestions. Only one sentence in the abstract and results still needs revision.

"This association increased with the age of the patients; RR =1.119, (95% CI 1.075; 1.065) for older than 60-year-old, RR=1.044 (95% CI 1.010-1.078) for ages 40-60 and RR= 1.000 (95% CI 0.977-1.023) for younger than 40-year-old.

There is still a problem with the 95% CI in the older persons!

**Response**:

**We kindly apologize for not fixing the typo: The true confidence interval for older persons is RR =1.119, (95% CI 1.075; 1.65) . We revised the manuscript accordingly. Page 2 and Page 7.**
